# Association between vaccination and preventive routines on COVID-19-related mortality in nursing home facilities: a population-based systematic retrospective chart review

**DOI:** 10.1017/S1463423622000640

**Published:** 2022-11-18

**Authors:** Lena Nilsson, Christer Andersson, Lisa Kastbom, Rune Sjödahl

**Affiliations:** 1 Department of Anaesthesiology and Intensive Care, and Department of Biomedical and Clinical Sciences, Linköping University, Linköping, Sweden; 2 Department of Orthopedics, Linköping University Hospital, Linköping, Sweden; 3 Department of Health, Medicine, and Caring Sciences, Linköping University, Linköping, Sweden; 4 Primary Health Care Center in Kisa, and Department of Health, Medicine, and Caring Sciences, Linköping University, Linköping, Sweden; 5 Department of Surgery and Department of Clinical and Experimental Medicine, Linköping University, Linköping, Sweden

**Keywords:** COVID-19, geriatrics, mortality, nursing homes, primary care, Vaccination

## Abstract

**Background:**

Older and frail individuals are at high risk of dying from COVID-19, and residents in nursing homes (NHs) are overrepresented in death rates. We explored four different periods during the COVID-19 pandemic to analyze the effects of improved preventive routines and vaccinations, respectively, on mortality in NHs.

**Methods:**

We undertook a population-based systematic retrospective chart review comprising 136 NH facilities in southeast Sweden. All residents, among these facilities, who died within 30 days after a laboratory-verified COVID-19 diagnosis during four separate 92-day periods representing early pandemic (second quarter 2020), middle of the pandemic (fourth quarter 2020), early post-vaccination phase (first quarter 2021), and the following post-vaccination phase (second quarter 2021). Mortality together with electronic chart data on demographic variables, comorbidity, frailty, and cause of death was collected.

**Results:**

The number of deaths during the four periods was 104, 120, 34 and 4, respectively, with a significant reduction in the two post-vaccination periods (*P* < 0.001). COVID-19 was assessed as the dominant cause of death in 20 (19%), 19 (16%), 4 (12%) and 1 (3%) residents in each period (*P* < 0.01). The respective median age in the four studied periods varied between 87and 89 years, and three or more diagnoses besides COVID-19 were present in 70–90% of the respective periods’ study population. Considerable or severe frailty was found in all residents.

**Conclusions:**

Vaccination against COVID-19 seems associated with a reduced number of deaths in NHs. We could not demonstrate an effect on mortality merely from the protective routines that were undertaken.

## Introduction

The COVID-19 pandemic has been a great challenge worldwide since early 2020. Older, frail people who suffer from several diseases are at high risk of dying from a COVID-19 infection (Hewitt *et al.*, 2020; Hollinghurst *et al.*, 2021; Pranata *et al.*, 2021). In many Western countries, residents in nursing homes (NHs) are overrepresented in death rates and account for 30%–40% of all deaths from COVID-19 while representing only approximately 0.5–1% of the population (Rolland *et al.*, 2021). NH care in Sweden is provided by two authorities in cooperation: regions and municipalities. Each NH has attending physicians employed at a regional primary healthcare centre, usually general practitioners or general practitioner specialist trainees. Nurses and other staff are municipality employees. An increasing number of older people in Sweden live in their own homes rather than in NHs (Swedish Institute, 2021), which has contributed to a situation in which people moving into NHs are usually the oldest and most frail. Median residency in a Swedish NH is 24 months (The National Board of Health and Welfare, 2020) and approximately 20% of the residents die within six months after moving into a NH (Ballin *et al.*, 2021). The reported percentage of overall excess deaths for Swedish NH residents in the Stockholm area was 167% in April 2020% and 46% in May 2020, compared to the mean during 2016–2019 (Strang *et al.*, 2020). Furthermore, it has been reported that the 30-day mortality rate after the diagnosis of COVID-19 was 39.9%, compared with 5.7% in matched controls (Ballin *et al.*, 2021).

In a review (Giri *et al.*, 2021) of challenges in NHs during the pandemic, five themes were identified: (1) patient characteristics including comorbidities, malnutrition and mental state; (2) staff – quality, staff levels, staff working in multiple NHs; (3) facility characteristics such as physical space, occupancy; (4) external factors regarding admissions and personal protection equipment and (5) asymptomatic transmission of COVID-19. Different strategies were undertaken to mitigate the impact of COVID-19. Cohorting and isolation are reported (Collison *et al.*, 2020; Gonzalez de Villaumbrosia *et al.*, 2020; Krone *et al.*, 2021) as well as strict visiting restrictions (Khan *et al.*, 2020). However, prolonged isolation including reduced staff-resident contact time and reduced social interactions predisposed the residents mostly being frail with multiple comorbidities to further physical and mental deconditioning (Iaboni *et al.*, 2020). Dementia made isolation and cohorting very difficult. NH personnel reported fear of contracting the virus and/or transmitting it to a loved one, and adequate supplies of personal protection equipment helped address many of these fears (McGilton *et al.*, 2020). To maintain social connection, numerous methods were observed, for example, via window or behind-glass visiting (Chow, 2021). Technology in NH care is not expanded and telehealth is not available in most NHs (Giri *et al.*, 2021).

NH residents were prioritized in the first phase of vaccination in many countries (Rolland *et al.*, 2021), including Sweden (Public Health Agency, 2021). This Swedish prioritization also included the NH staff. It is wellknown that the vaccine response in general is modulated by aging and frailty (Grubeck-Loebenstein *et al.*, 2009; Chan *et al.*, 2013). An impaired vaccination effect in NH residents, who are vulnerable individuals, would be problematic. It is therefore important to have a close follow-up on the effects of the COVID-19 vaccine in NHs to evaluate the vaccination effects in real life.

Generally speaking, NHs underwent a steep learning curve during the COVID-19 pandemic. Although the challenges were similar in different countries, they also differed depending on the context. Our research group has previously shown that during the first pandemic wave in 2020, death in Swedish home health care and NH facilities mostly affected the very oldest individuals with severe frailty and comorbidity (Nilsson *et al.*, 2021). Advance care planning was activated and reimplemented by the Regional executive office in our county early during the pandemic. At follow-up in June 2020, more than 90% of caretakers had individual advance care plans with special regard to serious respiratory disease documented in their health records in the county studied. To evaluate the effects of changed routines and vaccination, we found it interesting to study the Swedish context focusing on frail NH residents who in accordance with advance care planning were not transferred to hospital in case of a COVID-19 infection.

Our aim was to investigate in a defined population of residents cared for in NH facilities only, if preventive routines and vaccination against COVID-19 were associated with reduced mortality. Four separate and equally long time periods, representing different phases of the pandemic, were studied.

## Material and methods

### Study design and oversight

This population-based systematic retrospective chart review took place in Östergötland County Council, Sweden. The county has around 465 500 inhabitants and 136 NH facilities, with approximately 5000 residents in total. The exact number varies due to, for example, temporary or permanent reorganizations and deaths of residents. Also, the unique individuals living in NHs change as the median residency in a Swedish NHs is 24 months (The National Board of Health and Welfare, 2020). We studied consecutively all NH residents with a laboratory-confirmed COVID-19 diagnosis and who remained and subsequently died in the NH facilities within 30 days after the verified COVID-19 diagnosis during four separate periods of the pandemic. The first period from March 31 to June 30, 2020 starts with the first NH resident’s death on the NH (March 31, 2020) and represents the first pandemic wave with a high death rate connected to COVID-19 in NH facilities. Mortality and community spread of infection during the summer of 2020 was very low but increased again in the middle of October. To obtain a similar period of 92 days and to avoid the effect of vaccination that started the last week in December 2020, the middle of October was chosen as the starting point for the second review period, i.e. from October 15, 2020 to January 15, 2021. Consequently, the third 92 days-period was from January 16 to April 18, 2021, and the fourth period April 19 to July 20, 2021. Supplementary figure S1 displays the different pandemic waves, represented by the number of patients from the general population being hospitalized for COVID-19 in Östergötland County. The four studied periods are shown in the same figure.

Several protective measures were introduced early during the first pandemic wave in spring 2020 and adapted during the rest of the periods studied. Family members and others were prohibited from visiting NH facilities starting from April 1 2020 according to rules recommended by the Public Health Agency and regulated by the Swedish government. Great focus in the NHs was put on implementing preventive care hygiene routines including face masks, strict hand hygiene and use of gloves together with repetitive staff education. Access to personal protective equipment was initially challenging, but reliable availability subsequently followed.

When the second pandemic wave reached Östergötland County in October 2020, updated routines regarding preventive hygiene measures were in place, and access to personal protective equipment was safely secured in NHs. There had been organizational changes that, to some extent, made a cohort care of residents with COVID-19 in the NHs possible. Regulations on visits to NHs remained unchanged until October 1 2020, when visits were again allowed.

**Figure 1.**
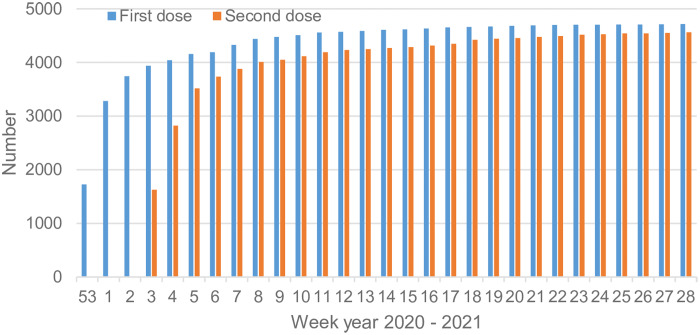
Accumulated number of vaccinated residents with one and two dose(s) in the studied 136 nursing homes housing approximately 5000 residents

Vaccination of NH residents against COVID-19 in Östergötland started in late December 2020 (week 53). This vaccination also included the NH staff involved in the care of NH residents. During three months (January to March 2021), approximately 90% of NH residents were administered two doses of BNT 162b2 mRNA vaccine, Pfizer/BioNTech Comirnaty vaccine. Figure 1 displays the vaccination progress in NH residents in Östergötland County Council, Sweden (personal communication).

The Swedish Ethical Review Authority approved the study.

### Data collection

The population-based study was performed as a systematic retrospective chart review. A regional population healthcare registry based on each individual’s unique social security number and reporting to the national authorities was used for initial case identification. From that registry, it was possible to assess the respective medical records and subsequently sort out individuals living in NHs and with a positive COVID-19 laboratory diagnosis within 30 days before death. Inclusion criteria were: all residents who died within 30 days of a positive COVID-19 diagnosis during the four periods described above. The detection of SARS-CoV-2 nucleic acid using the PCR technique or, less frequently, virus isolation confirmed the COVID-19 diagnosis. Merely clinical or radiological signs of COVID-19 did not qualify for inclusion in the study. NH residents that were referred to hospital care were excluded from the study as we aimed to analyze mortality in the cohort cared for only in a NH context.

Two senior experts in patient safety and health record review (RS, CA) retrospectively reviewed the health records of the included individuals to establish a pre-morbid level of function and to assess whether the cause of death was associated with COVID-19. Thus, from the records, patient-level data on demographic variables, clinical signs, comorbidity and frailty were collected.

### Definitions and outcomes

The number of each individual’s comorbidities, beside the COVID-19 diagnosis, was graded into three groups: 0–1, 2, and 3 or more diagnoses, respectively (Nilsson *et al.*, 2021).

The combined assessment of performance status and frailty was adapted to the health record review method (Nilsson *et al.*, 2021) and described the individual’s status during the months preceding the COVID-19 disease. We used a modification of the World Health Organization/Eastern Cooperative Oncology Group (WHO/ECOG) performance status and frailty score according to Rockwood *et al.* (2005) and classified the individuals into four groups (Nilsson *et al.*, 2021):

- None or mild frailty: no restrictions in daily life
- Moderate frailty: mobile and independent but unable to handle physically demanding activities or work
- Considerable frailty: able to perform activities of daily living but confined to bed or chair, over half the time being awake
- Severe frailty: unable to perform activities of daily living and/or confined to bed or chair; dementia requiring care in NH

In the assessment of the cause of death, a thorough analysis was first performed by one of the reviewers, followed by discussion until a consensus was reached between the two reviewers. The individuals’ symptoms together with the time between the confirmed diagnosis and death was considered, but whether they had received the vaccine was not taken into account as we strived for a neutral evaluation and also could not from the record review estimate the specific individual’s effect of the vaccination. We identified three groups: (1) COVID-19 was the fully dominant cause; (2) COVID-19 contributed in conjunction with other comorbidities, such as heart failure, pulmonary disease or dementia and (3) death was most likely caused by a disease other than COVID-19, such as cancer in the terminal phase (Nilsson *et al.*, 2021; Spreco *et al.*, 2022).

### Statistical analysis

The descriptive data are presented using the median (range) or in numbers (*n*) and percentages (%). For comparisons, the quantitative data were analyzed using Kruskal–Wallis ANOVA and the categorical data using the Pearson chi-square test. A *P*-value <0.05 was regarded as statistically significant.

## Results

In the studied 136 NH facilities with approximately 5000 residents, the number of deaths in residents with laboratory-confirmed COVID-19 was 104 and 120, respectively, during the first and second pre-vaccination periods, and 34 and 4, respectively, during the third and fourth post-vaccination periods (*P* < 0.001). Significant differences were found between the first and second periods and the third and fourth periods (all *P* < 0.001) and also between the third and fourth periods (*P* < 0.001). In total, 45 deceased residents (12, 12, 18, 3 in the respective four periods) were excluded from the study, as they were referred from the NH to hospital and died while still being hospitalized. Demography, comorbidity and frailty of the studied population of deceased NH residents are shown in Table 1.

**Table 1.** Demography, comorbidity and frailty of the diseased nursing home residents in the four studied phases

	First period 03/31/20–06/30/20 (104 deaths)	Second period 10/15/20–01/15/21 (120 deaths)	Third period 01/16/21–04/18/21 (34 deaths)	Fourth period 04/19/21–07/20/21 (4 deaths)	*P*
Age (median [range])	88 (69–106)	89 (60–100)	89(69–100)	87.5(82–90)	0.92
Male/female (%)	44/56	42/58	38/62	75/25	0.55
Comorbidity^*^ *n* (%)					
≤1 diagnose	2 (2)	6 (5)	4 (12)	0 (0)	0.07
2 diagnoses	8 (8)	13 (11)	6 (18)	1 (25)	
≥3 diagnoses	94 (90)	100 (83)	24 (71)	3 (75)	
Frailty					
None, mild	0 (0)	0 (0)	0 (0)	0 (0)	<0.01^**^
Moderate	0 (0)	0 (0)	0 (0)	0 (0)	
Considerable	4 (4)	25 (21)	5 (15)	0 (0)	
Severe	100 (96)	95 (79)	29 (85)	4 (100)	

* Comorbidity could not be assessed for one of the residents in the second period.

** Significance calculated between considerable and severe frailty.

**Figure 2.**
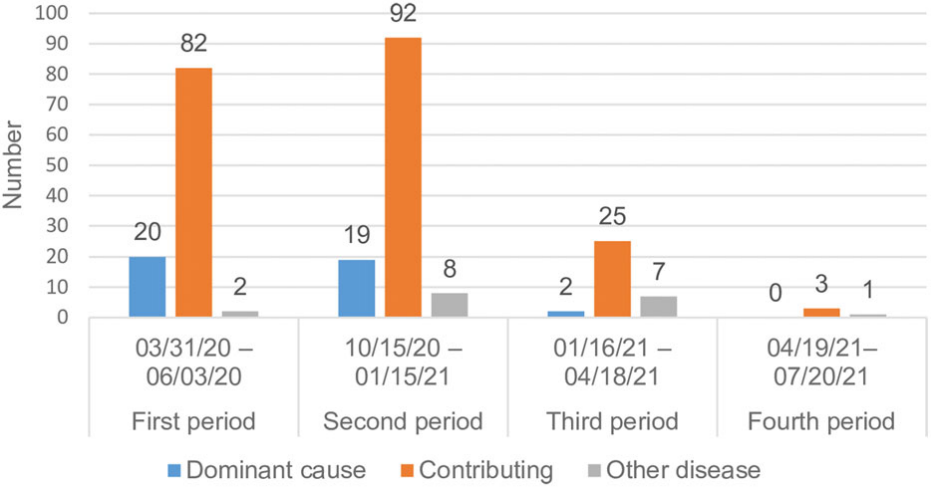
Assessed contribution of COVID-19 to the cause of death in nursing home residents

The role of COVID-19 as contributor to the cause of death in NH residents with laboratory-confirmed COVID-19 differed between the periods (*P* < 0.01, Figure 2). The incidence of COVID-19 as the dominant cause of death decreased during the third and fourth periods. For all the four periods together, COVID-19 was mostly assessed as a contributing cause of death.

Besides pulmonary disease being more prevalent among the residents in the second period, there were no differences in the most common contemporary diagnoses among the studied NH residents (Table 2).

**Table 2.** Contemporary diagnoses beside COVID-19 in the study population of diseased residents in nursing home facilities

	First period 03/31/20–06/30/20 (104 deaths) *n* (%)	Second period 10/15/20–01/15/21 (120 deaths) *n* (%)	Third period 01/16/21–04/18/21 (34 deaths) *n* (%)	Fourth period 04/19/21-07/20/21 (4 deaths) *n*	*P*
Dementia	63 (64)	76 (63)	21 (62)	2 (50)	0.94
Heart disease	54 (52)	70 (58)	20 (59)	2 (50)	0.69
Diabetes	25 (24)	36 (30)	8 (23)	1 (25)	0.64
Stroke	14 (13)	13 (11)	5 (15)	0 (0)	0.77
Pulmonary disease	6 (6)	24 (20)	4 (11)	1 (25)	0.02
Renal failure	10 (10)	16 (13)	5 (15)	1 (25)	0.52
Cancer	4 (4)	6 (5)	3 (9)	0 (0)	0.92

One individual can have several diagnoses.

Among the individuals who died during the second period (ending January 15, 2021), three had received their first vaccination dose (December 27, 2020 [*n* = 2] and January 5, 2021 [*n* = 1]). None had been given the second dose.

During the third period, 21 (62%) of the 34 individuals who died had undergone vaccination: 15 with one dose and six with two doses (Table 3). Of these individuals, 11 died 3 weeks or more after the first vaccination dose and three of them died more than 3 weeks after the second vaccination dose. COVID-19 was assessed as the dominant cause of death in four individuals, two having had one vaccination dose and two had not been vaccinated.

**Table 3.** Assessed contribution of COVID-19 to the cause of death in relation to vaccination status during the third and fourth periods

Contribution of COVID-19 to the cause of death	Vaccination one dose	Vaccination two doses	No vaccination
Third period (34 deaths)			
Dominating cause (*n* (%))	2 (6)	0 (0)	2 (6)
Contributing cause (*n* (%))	12 (35)	3 (9)	8 (24)
Other disease (*n* (%))	2 (6)	2 (6)	3 (9)
Fourth period (4 deaths)			
Dominating cause (*n* (%))	0 (0)	0 (0)	0 (0)
Contributing cause (*n* (%))	1 (25)	1 (25)	1 (25)
Other disease (*n* (%))	0 (0)	1 (25)	0 (0)

During the fourth period, one of the individuals who died had not undergone vaccination (Table 3).

## Discussion

In the third and fourth post-vaccination periods of the pandemic, there was a significant reduction in the death rate, indicating a protective effect of the vaccine against mortality in an especially vulnerable group of individuals with laboratory-verified COVID-19. In contrast, the number of deaths remained unchanged in both the first and second periods, although several educational and organizational measures had been undertaken in order to reduce the spread of the infection. No differences in age, gender or comorbidity among the deceased individuals were identified between the four separate periods.

We regard the significant reduction in mortality during the third and fourth periods as an effect of the vaccination program, as this reduction took place while the incidence of COVID-19 was still high in the total population in the society, with a peak value of 1000 per 100 000 inhabitants in late April 2021 (Region Östergötland, personal communication). Furthermore, we noted a relative increase in diseases other than COVID-19 as the cause of death, indicating a positive effect of vaccination. This study contributes to important real-world learning from population-based trials on the effects of vaccination in vulnerable groups. In Ontario, Canada, the relative reduction in COVID-19 mortality in vaccinated long-term care home residents was 96% after 8 weeks and considerably exceeded the reduction seen in the unvaccinated control population (Brown *et al.*, 2021). Positive post-vaccination results are also reported from NHs in the USA with a reduction in resident deaths (incidence rate ratio [IRR] 0.45 (Domi *et al.*, 2021), reduced risk for a positive SARS-CoV-2 test (relative risk ratio 0.37) (Rudolph *et al.*, 2021) and reduced rates of COVID-19-associated deaths (Mor *et al.*, 2021). Isolation to prevent infection is difficult in NH residents, as they are often old and frail and thus are dependent on others for their personal care. What is more, dementia is frequently present in NH residents, and keeping these individuals isolated in their rooms could be extra challenging. This strengthens the importance of studying vaccination effects in real life in this vulnerable group.

In many countries, the first phase of vaccination focused on NH residents (The National Board of Health and Welfare, 2020), as the COVID-19 pandemic disproportionately impacts frail, older people. Mortality increases exponentially with age over 50, and most fatalities have occurred in those over 80 years (Bonanad *et al.*, 2020; Kang and Jung, 2020). In Swedish NHs, 30-day mortality was seven times higher in residents with COVID-19 than in matched controls without COVID-19 (Ballin *et al.*, 2021). Furthermore, NH residents had a four times higher risk of COVID-19 mortality than older adults living in independent housing (Brandén *et al.*, 2020). However, frailty does not seem to be adequately considered regarding safety and efficacy in studies of COVID-19 vaccines (Andrew *et al.*, 2021; Soiza *et al.*, 2021). Early preliminary data have suggested that the older population included in some vaccine trials might not represent the older population dying from COVID-19, including the frail and multimorbid residents of NHs (Antonelli Incalzi *et al.*, 2021). Bearing these shortcomings in mind, it is important that clinical, real-world studies on these vulnerable groups are rapidly shared.

In our studied population, three-quarters of the diseased individuals in the third period were vaccinated, with the majority (70%) having received one dose of the vaccine more than three weeks before their death. Thus, vaccination did not seem to fully prevent from being infected by SARS-CoV-2. The number of studied individuals in the third and fourth periods is limited, and no firm conclusions concerning vaccination breakthrough can be drawn.

In the assessment of the cause of death, the group “COVID-19 contributed in conjunction with other comorbidities” dominated in all four periods. This reflects the substantial comorbidity in this vulnerable group of individuals. Frailty is associated with mortality in older patients with COVID-19 (Aw *et al.*, 2020) and was found to predict mortality better than age or comorbidity in patients hospitalized for COVID-19 (Hewitt *et al.*, 2020). Therefore, a frailty scale could be used for risk stratification. In the present study, a modification of the scoring was needed to suit the retrospective health record review approach, and frailty was categorized into only four groups.

We found a significant difference regarding the difference in pulmonary disease between the four studied periods. In relation to the multimorbidity in the group dominated by other morbidities, we regard this finding as of minor importance (Hussien *et al.*, 2021).

The spread of the infection from asymptomatic healthcare staff is one possible explanation for why the COVID-19 disease entered the NHs. It has been estimated that at least 50% of new SARS-CoV-2 infections in a community originated from asymptomatic individuals (Johansson *et al.*, 2021). In parallel to the vaccination of NH residents, healthcare staff was vaccinated against COVID-19. This was voluntary, and the willingness to vaccination was perceived as high. However, no registration of vaccination coverage is allowed in Sweden. Preclinical studies of mRNA candidate vaccines have shown a persistent presence of the virus in nasal swabs, despite individuals being clinically asymptomatic (Bleier *et al.*, 2021). In a US study, consecutive preprocedural routine tests for SARS-CoV-2 in almost 40 000 patients were retrospectively analyzed to assess the impact of vaccination. Ten days or more after one vaccination dose of an mRNA COVID-19 vaccine, the risk of asymptomatic COVID-19 significantly decreased (RR 0.21) (Tande *et al.*, 2022). A positive vaccine effect on asymptomatic infection has also been reported in England in a study on healthcare workers (Hall *et al.*, 2021).

We could not demonstrate any effect on the mortality in the second period during the autumn of 2020 from the preventive measures that were implemented during and after the first study period in the spring of 2020. The personnel were continuously trained and provided with access to personal protective equipment, and separate cohort care facilities were at hand. Others have speculated that asymptomatic staff, including clinicians who come in and out of the facility, are likely to be the source of infection (Ouslander and Grabowski, 2020). These authors suggest several important ways to reduce the risk of an escalating infection in NH facilities: telehealth visits to prevent unnecessary hospital transfers, isolation for 10–14 days of all new admissions, immediate isolation and viral testing in connection with almost any acute change in health and improved advance care planning by obtaining and documenting care-limiting orders. In the present study, prohibition against visiting NHs was instituted only during the first period. Telehealth consultations were well established before the pandemic and were thus frequently used. Advance care planning, including proactive plans in case of deterioration, was part of a systematic approach initiated by the management and already in place early during the first period. Advance care plans were present in over 90% of the residents in our study population.

The COVID-19 variants that dominated in our region during different periods were alfa from the beginning of the pandemic to July 2021. The delta variant was detected in late May 2021, and in the beginning of July 2021, it dominated over the alfa variant. The delta variant fully dominated until it was rapidly replaced by the omicron variant in late December 2021. This means that during our studied four periods, the alfa variant was the dominating one, with the delta variant gradually increasing during the fourth period.

The present study is strengthened by the fact that it comprises all NHs in the region and describes both frailty and comorbidity. All COVID-19 diagnoses were laboratory-verified, also after vaccination. The health record review methodology, and the fact that the reviews were performed by two senior experts in patient safety and health record review, provide more detailed information on health and frailty than a register-based approach does.

There are some limitations to this study. Although it comprised all deceased residents with a positive COVID-19 diagnosis in a defined group of NHs with about 5000 residents, the number included in the study was limited. There are also important confounding factors that were not possible to study by using the retrospective record method, for example, personnel compliance to preventive hygiene rules, de facto use of protective equipment, personnel vaccination compliance and frequency of visitors. The assessment of the cause of death was, at times, limited by sparse documentation in the medical records. We had no access to separate documentation from personnel at the NH facilities. Another limitation is the retrospective data collection. Apart from a COVID-19 test, radiological and laboratory investigations were seldom undertaken as part of advance care planning for the resident, advocating continued care in the NH facility. No calculation of inter-rater reliability was done. During the first period, the knowledge also grew that discrete symptoms, such as anorexia and general deterioration, might be the only signs of COVID-19. Therefore, it may be that not all possible COVID-19 patients were included in the study early during the first period, as testing may not have been not performed. Likewise, there was a risk that discrete COVID-19 symptoms in vaccinated individuals in the third and fourth periods were not tested for the virus.

## Conclusions and implications

Vaccination against COVID-19 seems associated with reduced mortality in NH residents characterized by advanced age, comorbidity and frailty. Our results have implications for healthcare policymakers as it, together with other studies, strengthens the early prioritizing of vaccination for this vulnerable group. We could not demonstrate an effect merely of the implemented protective routines on mortality. However, hygiene routines and protective equipment form a necessary base in the care of elderly and frail individuals. Focus on these measures should be maintained, and our results cannot be taken as an excuse for deviations from the routine.

## Supplementary material

For supplementary material accompanying this paper visit http://doi.org/10.1017/S1463423622000640. click here to view supplementary material
