# Supplementary material for: Association between vaccination and preventive routines on COVID-19-related mortality in nursing home facilities: a population-based systematic retrospective chart review
Source: Prim Health Care Res Dev. 2022 Nov 18;23:e75. doi: 10.1017/S1463423622000640 (PMC9706376; doi:10.1017/S1463423622000640)
Supplement: Supplementary file 1 [file S1463423622000640sup001.docx]

Supplementary Figure S1


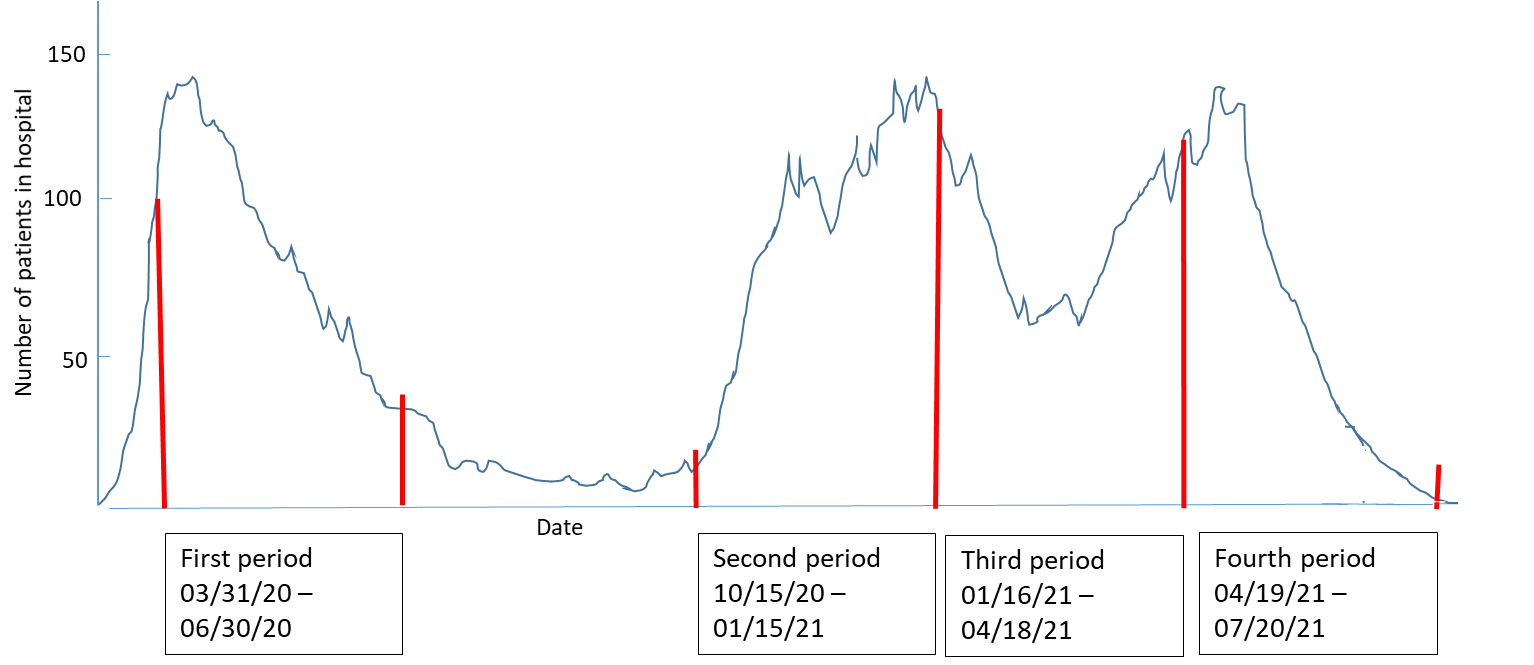


The studied periods of COVID-19 related death in nursing homes and the pandemic waves in the studied region represented by hospitalized COVID-19 patients from the total society population.
